# Morphological entropy encodes cellular migration strategies on multiple length scales

**DOI:** 10.1038/s41540-024-00353-5

**Published:** 2024-03-07

**Authors:** Yanping Liu, Yang Jiao, Qihui Fan, Xinwei Li, Zhichao Liu, Dui Qin, Jun Hu, Liyu Liu, Jianwei Shuai, Zhangyong Li

**Affiliations:** 1 Department of Biomedical Engineering, Chongqing University of Posts and Telecommunications, Chongqing, China; 2 Chongqing Key Laboratory of Big Data for Bio Intelligence, Chongqing University of Posts and Telecommunications, Chongqing, China; 3 Materials Science and Engineering, Arizona State University, Tempe, AZ USA; 4 Department of Physics, Arizona State University, Tempe, AZ USA; 5 Beijing National Laboratory for Condensed Matter Physics and CAS Key Laboratory of Soft Matter Physics, Institute of Physics, Chinese Academy of Sciences, Beijing, China; 6 Department of Neurology, Southwest Hospital, Army Medical University, Chongqing, China; 7 Chongqing Key Laboratory of Soft Condensed Matter Physics and Smart Materials, College of Physics, Chongqing University, Chongqing, China; 8 Department of Physics, Xiamen University, Xiamen, China; 9 Fujian Provincial Key Laboratory for Soft Functional Materials Research, Xiamen University, Xiamen, China; 10 Wenzhou Institute, University of Chinese Academy of Sciences, Wenzhou, China

**Keywords:** Biophysics, Biological physics

## Abstract

Cell migration is crucial for numerous physiological and pathological processes. A cell adapts its morphology, including the overall and nuclear morphology, in response to various cues in complex microenvironments, such as topotaxis and chemotaxis during migration. Thus, the dynamics of cellular morphology can encode migration strategies, from which diverse migration mechanisms can be inferred. However, deciphering the mechanisms behind cell migration encoded in morphology dynamics remains a challenging problem. Here, we present a powerful universal metric, the Cell Morphological Entropy (CME), developed by combining parametric morphological analysis with Shannon entropy. The utility of CME, which accurately quantifies the complex cellular morphology at multiple length scales through the deviation from a perfectly circular shape, is illustrated using a variety of normal and tumor cell lines in different in vitro microenvironments. Our results show how geometric constraints affect the MDA-MB-231 cell nucleus, the emerging interactions of MCF-10A cells migrating on collagen gel, and the critical transition from proliferation to invasion in tumor spheroids. The analysis demonstrates that the CME-based approach provides an effective and physically interpretable tool to measure morphology in real-time across multiple length scales. It provides deeper insight into cell migration and contributes to the understanding of different behavioral modes and collective cell motility in more complex microenvironments.

## Introduction

Cell migration plays a vital role in the normal development of tissues or organs, including wound healing^1–3^, immune response^4^, and embryogenesis^5^. In addition, many human diseases are mainly dominated by dysregulated cell migration, such as cancer invasion and metastasis^6,7^.

Typically, cells migrating in complex microenvironments are regulated by environmental cues^8^ and intracellular signaling pathways^9^, resulting in diverse modes of cell migration^10–12^. For example, chemotaxis mediated by diffusible cues^13^, haptotaxis in response to surface-bound chemical cues^14^, and durotaxis in response to differences in substrate stiffness^15^. More specifically, MDA-MB-231 cells exhibit a limit cycle in two-state micropatterns, whereas MCF-10A cells show excitable bistable dynamics^16^. Intriguingly, cells reversing, following, and sliding past each other upon collision have been observed in these micropatterns^17^. Furthermore, oriented collagen fibers in the microenvironment can stabilize cellular protrusions and additionally guide 3D cell migration^18^. During directed migration, the corresponding persistence is exponentially correlated with the migration velocity, and this typical relationship is dominated by actin flows and regulated by the Arp2/3 complex that supports lamellipodia extension^19–21^. Besides the migration dynamics, cells also exhibit distinct morphological dynamics in response to different external cues. Particularly, cell nuclei are stretched to overcome the steric hindrance caused by physical constraints^22^, which is strongly correlated with nuclear envelope stretch-sensitive proteins^23^. Since both cell morphology and migration modes are the consequences of a combination of extracellular cues and intracellular signals, they are closely related to each other. For example, single-cell migration has been classified into mesenchymal and ameboid modes. The former is dominated by the actin polymerization that pushes the plasma membrane forward and exhibits several features, including a strong dependence on adhesion to the extracellular matrix (ECM), an elongated morphology in 3D environments, and actin-based protrusions at its leading edge; whereas the latter depends mainly on actomyosin contractility and exhibits a more rounded morphology, especially undergoing constant changes in shape due to the rapid extension and retraction of membrane protrusions^24–26^.

To decipher the migration mechanisms based on the most vivid cell morphology, many novel researches have been carried out^27^. For example, cell morphology neural networks are constructed to identify subcellular compartments and the cell types of neuron reconstructions^28^. Machine learning is also employed to classify cell shapes into distinct phenotypes, indicating that morphological phenotypes controlled by ECM mechanics and Rho/ROCK-signaling facilitate cancer cell navigation through the non-uniform ECM^29^. Recent work shows that morphological classes of single cell-derived clones derived from 216 features of the cell and nucleus can predict unique tumorigenic and metastatic potentials in vivo using unsupervised clustering analysis^30^. Moreover, shape fluctuations of the chromatin globule surface and nuclear envelope are driven both thermally and actively, with decreasing amplitudes serving as a reliable cell cycle stage indicator^31^. In the previous work, we also developed a quantitative approach that contains five shape parameters, i.e., the perimeter^2^-to-area ratio (${r}_{{pta}}$), the standard deviation of the relative boundary-to-center distances (${d}_{{std}}$), the protrusion height ($h$), the height-to-width ratio (${r}_{{htw}}$), and the combination of ${h\& }{r}_{{htw}}$, to depict the morphological characteristics of cell spheroids and verified the ability of the DDR1 inhibitor 7rh to weaken the invasion of single cells^32^. Even so, the approach may have limitations in clarifying relevant biophysical interpretations due to the multiple shape parameters. In addition, some scalar descriptors including roughness, shape factor, and curvature, are often used to characterize the morphology of cells and nuclei^33–35^, and the corresponding analysis aims to capture more information to study and distinguish biological states closely related to morphological dynamics^36^. Among these methods, the widely used CellProfiler^37^ and MorpholibJ^38^ (a plugin for ImageJ) allow us to extract a large number of features and further reveal the differences across varying cell populations.

Taken together, there is increasing evidence that the study of cell morphology and its relationship to cell functions and migration modes can provide more insight into the mechanisms underlying cell migration. However, the approaches or tools used still have three shortcomings: i) the approaches are mainly used to analyze the morphological changes of single cells or nuclei, and it is unknown how they behave when used to analyze other objects such as cell spheroids^33–35^; ii) the approaches involve many features that allow us to capture enough information of the shape, but these numerous descriptive features could limit the biophysical interpretation of shape changes^32,37,38^; iii) the approaches are mainly developed based on machine learning algorithms and large amounts of morphological data, which may be limited in analyzing time-varying morphological features^27,29,30^. Therefore, quantifying morphological features of different objects in real-time using a simple and efficient approach becomes a major challenge.

Here, we introduce a theoretical metric that combines morphological analysis and Shannon entropy to depict the morphological dynamics of some objects that are mediated by complex physical or biochemical cues. Note that the Shannon entropy introduced has a physical interpretation akin to the Gibbs entropy for thermal systems. It reflects the degree of order or randomness of cellular migration, with a value of 1 indicating purely diffusive migration (i.e., the most “random” dynamics) and a value of 0 indicating purely ballistic dynamics (i.e., the most “ordered” dynamics). We find that for different length scales of cell data, including nuclei, single cells, and cell spheroids, the approach can accurately measure the changes in morphology, especially the angular and radial features. By analyzing the time-dependent CME components, we gained some insights encoded in the morphology changes, including the dynamics of the nucleus in overcoming the steric hindrance of the ECM, the interactions of MCF-10A cells migrating on top of a 3D collagen gel, and the transition of tumor spheroids from proliferation to invasion under the regulation of the DDR1 inhibitor 7rh. Thus, the CME metric allows us to explore cell migration mechanisms in pathophysiological environments, such as cancer and other physiological conditions.

## Results

### Biophysical interpretations of the CME metric

**Fig. 1:**
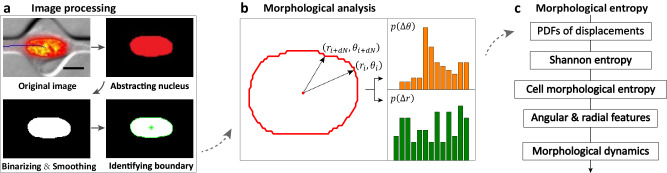
Flowchart of CME construction. **a** Representative workflow of image processing. The original image was taken with permission from the work^22^ and shows that the MDA-MB-231 cell nucleus in red is located in a chamber. Scale bar, 10 µm. **b** Morphological analysis of the sample in the polar coordinate system (PCS). The green and orange bars represent probability density functions (PDFs) of radial and angular displacements, respectively. **c** Derivation of the CME based on PDFs and Shannon entropy.

**Fig. 2:**
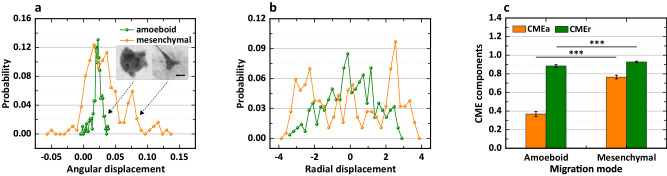
Biophysical interpretations of the CME metric. **a** PDFs of the angular displacement of cell morphology. The inset shows representative single-cell migration modes, adapted from the work^58^ under CC–BY license. Scale bar, 10 µm. **b** PDFs of the radial displacement. **c** CME components of the two types of migration modes. Data are presented as mean ± sd (standard deviation); *n* = 10 and 8 for ameboid and mesenchymal modes, respectively; $* * * p$ < 0.001; *t*-test; and the lag ${dN}$ is 4.

To clearly illustrate the biophysical interpretations of the CME metric, we analyzed the morphology of two types of single-cell migration following the procedure described in Fig. 1, i.e., ameboid and mesenchymal modes of migration (see the inset in Fig. 2a), and the corresponding results are exhibited in Fig. 2. Evidently, the PDFs of the angular displacement for the two types of morphologies possess the different trends as a whole, i.e., the probability values for the ameboid mode are located in the small interval of 0.0–0.05, while the values for the mesenchymal mode cover an extensive range of -0.05 to 0.15 (Fig. 2a). Consequently, the difference results in a narrower PDF of angular displacement for the ameboid mode compared to the mesenchymal mode, which theoretically indicates that the blebs (or protrusions) of the ameboid mode are more uniformly distributed on the angular direction. It’s worth noting that the term “narrower” is used when the PDF deviates from a uniform distribution. Similarly, the PDF of the radial displacement for the ameboid mode is narrower than that for the mesenchymal mode (Fig. 2b), showing that the blebs of the ameboid mode are more uniformly distributed in the radial direction. In addition, the CME components (i.e., CMEa for angular and CMEr for radial features) of the two types of morphologies also exhibit significant differences, namely, the CMEa for the ameboid mode is significantly smaller than that for the mesenchymal mode, and the CMEr for the former is also slightly smaller than that for the latter, but with statistical significance ($* * *\, p$ < 0.001) (Fig. 2c). Here, the values of the CME components are mainly determined by the natural features of the cell morphology, so it is very possible that this metric could be used to distinguish the modes of cell migration. To further explore the performance of the CME approach, we additionally analyzed multiple and single lamellipodia with similar features, which differs from the significant differences between ameboid and mesenchymal modes. The results agree well with our assessment and further confirm the effectiveness of the CME approach in capturing some subtle differences in morphology. See Supplementary Fig. 1 for a more detailed discussion.

Taken together, the CME can be used to measure the angular and radial features of a given morphology, and the more uniformly distributed the features are, the smaller the value of the CME. To better understand the relationship above, we conclude the following two aspects: (i) if there are multiple non-obvious features (e.g., blebs) in the angular direction, we could consider that they are more uniformly distributed on this direction and result in a narrower PDF (smaller CMEa); (ii) if there are significant differences in the features (e.g., protrusions) on the radial direction, we also consider that they are less uniformly distributed on this direction and result in a broader PDF (larger CMEr). In other words, the CMEa and CMEr describe the heterogeneity of the angular and radial features, respectively.

### Morphological dynamics of MDA-MB-231 cell nucleus squeezing through a micro-structured array

In the previous subsection, we have clearly clarified the biophysical interpretations of the CME metric based on the cell morphologies of the ameboid and mesenchymal modes. Now, we further apply the CME approach to investigate the changes in nuclear morphology as the cell squeezes through spatial constraints. Here, the images of cell nuclei (length scale, ~ 20 µm) are taken from the videos published by Fabry et al.^22^, in which the authors studied 3D migration in a confined environment of varying stiffness. See more exciting results in the works^22,39^.

#### Features of the channel array characterized by the CME approach

**Fig. 3:**
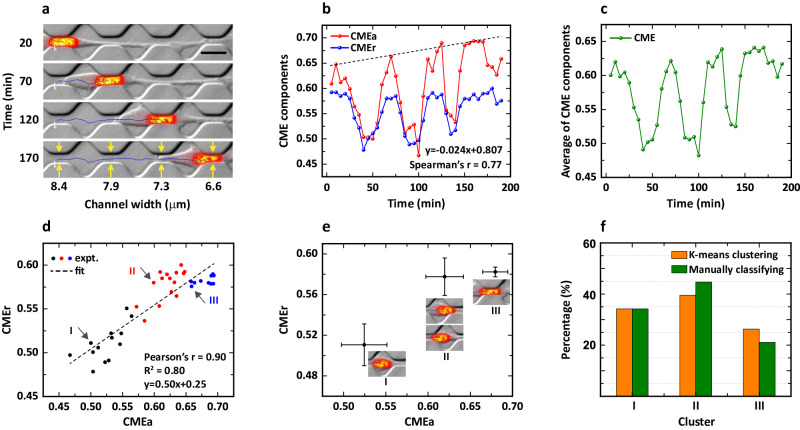
Morphological dynamics of the MDA-MB-231 cell nucleus in a micro-structured channel array. **a** Breast cancer cells migrate in a microstructure consisting of sequential channels and chambers, adapted with permission from the work^22^. Nuclei are stained with Hoechst and shown in red. Scale bar, 20 µm. **b** CME components of angular (red) and radial (blue) displacements as a function of time. The dotted line is a guide for the eye, and the lag ${dN}$ is 2. **c** Average of the CME components in **b** as a function of time. **d** The scatter of CMEr *vs*. CMEa. The dotted line is a linear fit to the experimental data, divided into three clusters (see black, red, and blue dots) using K-means clustering. **e** Three states are indicated by the scatter of CMEr *vs.* CMEa. Data are presented as mean ± s.d. **f** Percentage of scatter in each cluster obtained by K-means clustering (orange bars) and manually classifying (green bars).

In this subsection, we first convert the video of cell migration into a series of images, and analyze the time-lapse images using the CME approach, and the corresponding results are exhibited in Fig. 3. It clearly indicates that a cell nucleus squeezes through a narrow channel (as marked by the yellow arrows) at different times, and the nucleus is “rod-like” due to the physical constraints with a gradually decreasing width from 8.4 to 6.6 µm (Fig. 3a). Furthermore, the migration speed seems to be qualitatively stable, as the nucleus passes through the chamber at the time interval of ~ 50 min, as indicated by the labels on the vertical axis of Fig. 3a. In terms of quantitative analysis, both CME components (CMEa and CMEr) possess the exact characteristics of “peak and valley” (Spearman’s coefficient = 0.77), i.e., four peaks and three valleys, which visibly show the effects of these channels and chambers on the cell nucleus, respectively (Fig. 3b). In addition, the CME components also behave differently, i.e., the values of peak and valley for CMEa gradually increase, as indicated by the dotted line. However, the values of the peak for CMEr are almost stable around 0.58, which also differs significantly from the increase in those of the valley. The changing trends of the CME components above further illustrate that CMEa and CMEr respond differently to the same external cues in the ECM, which may reflect the intrinsic properties of the nucleus to some extent, such as the stiffness that is mainly dominated by nuclear envelope stretch-sensitive proteins^23^. Here, the changing trends could also be captured by the aspect ratio (AR) metric that is used to measure the elongation (see Supplementary Fig. 4a, b), since the changes in morphology of the nucleus are dominated by elongation. To assess the changes in the cell nucleus as a whole, we also average the CME components and obtain the resulting averaged CME, which follows a similar trend to that of CMEa (Fig. 3c).

Although the CME components have distinct differences, CMEa is still strongly correlated with CMEr (Pearson’s coefficient = 0.90). For example, CMEr gradually increases as CMEa increases and this relationship could be fitted well by a linear variation “y = 0.50x + 0.25” (R^2^ = 0.80), as indicated by the scatter in Fig. 3d. To explore the potential causality of this correlation, we also compute the cross-correlation between the radial $\Delta {r}_{j}$ and angular $\Delta {\theta }_{j}$ displacements taking tumor spheroids as an example, see Supplementary Fig. 3 for more details. According to the features of the scatter, we further classify the scatter into three clusters (labeled by I, II, and III) using the K-means clustering algorithm^40^. The averaged values for each cluster are plotted in Fig. 3e, where the error bars denote the s.d. for the two CME components. It can be seen that CMEa increases significantly from 0.52 to 0.62 and then to 0.68, while CMEr first increases from 0.51 to 0.58 and then remains stable around 0.58, forming three clusters for CMEa and two clusters for CMEr. On the one hand, the three clusters for CMEa in Fig. 3e could be perfectly explained by three migration states in the micro-structured array: (i) cluster I shows that the cell nucleus is located at a chamber and possesses a smaller CMEa as it has more space to recover from the highly confined state; (ii) cluster II shows that the cell nucleus is entering (or exiting) the channel and the confinement gradually increases (or decreases); (iii) cluster III illustrates that the cell nucleus is squeezing through the channel and has a larger CMEa because of the stronger physical confinement. On the other hand, the two clusters for CMEr could be utilized to identify different structures, i.e., the large CMEr corresponds to the channel while the small CMEr corresponds to the chamber. In addition, the two aspects above also directly reflect that CMEa is more sensitive to spatial confinement compared to CMEr.

#### Estimation of the steric hindrance of the channel array

Finally, we counted the number of scatter points in each cluster and plotted the histogram in Fig. 3f. The results show that the percentages for the clusters I–III are 34.2%, 39.5%, and 26.3%, respectively, which closely correlate with the results of 34.2%, 44.7%, and 21.1% obtained by manually observing the images of the cell nucleus and then classifying them into their respective clusters. Due to the unchanged sampling time $\Delta t$ = 5 min, the percentage here could also be considered as (or equivalent to) the dwell time of a cell nucleus in a unique structure. According to the array with three chambers (23 µm in length) and four channels (18 µm in length) through which the nucleus travels, we roughly estimate the time the nucleus spends in the chambers and channels.

The corresponding ratio is theoretically equal to (3*23)/(4*18) = 0.96, i.e., the ratio of chamber length to channel length, assuming that the nucleus moves through the channel array at a constant speed. Specifically, the time spent in cluster I is actually 1.3 times greater than the time in cluster III, and the time in cluster II is 1.5 times greater than the time in cluster III. Additionally, the time in cluster I is 0.52 times greater than the total time in clusters II and III. According to the above comparisons, we could deduce three important aspects: i.e., (i) the value of 1.3 indicates that the unconstrained nucleus (cluster I) takes more time than the fully constrained nucleus (cluster III), (ii) the value of 1.5 indicates that the partially constrained nucleus (cluster II) also takes more time than the fully constrained nucleus (cluster III), and (iii) the value of 0.52 indicates that the narrow channels impede the movement of the nucleus compared to the chambers. Overall, the geometric confinement within the ECM can cause deformation of the cell nucleus and further hinder cell migration to some extent.

### Emerging interaction of MCF-10A cells migrating on top of a 3D thick collagen gel

To validate the utility and efficiency of the CME approach, we further investigate the morphology of MCF-10A cells migrating on top of a 3D ECM based on collagen I hydrogel (i.e., an 800-µm thick layer of collagen gel). This model can mimic an in vivo quasi-3D system, such as cells moving at the interface of tissues. Furthermore, the fibrous structure of the collagen gel helps to support long-range force propagation, which induces strong cell-ECM mechanical coupling and directs highly correlated cell migration (length scale, ~120 µm). See our previous works^41,42^ for more details. However, it remains unclear how cell morphology changes during correlated migration.

#### Alternating changes in the morphology of a pair of cells

**Fig. 4:**
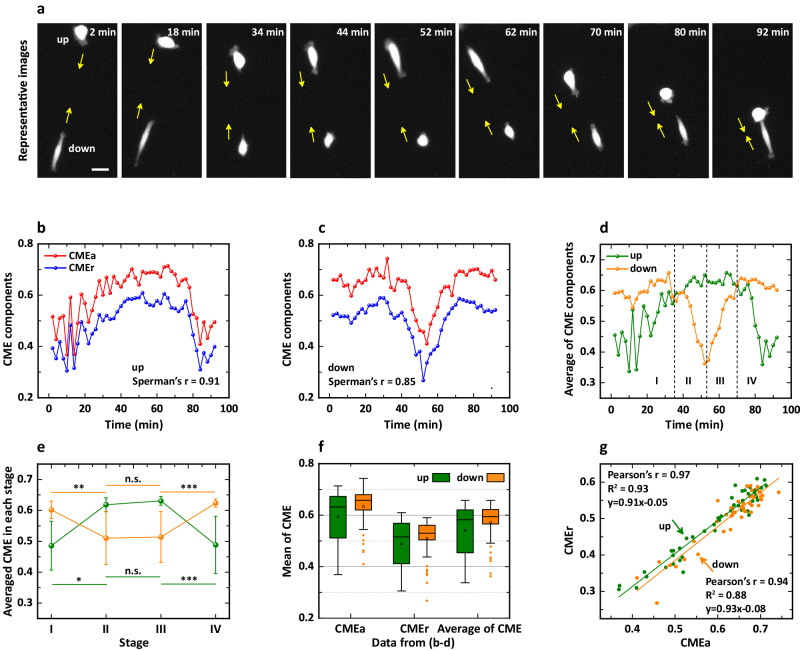
Emerging interaction of MCF-10A cells migrating on top of a thick 3D collagen gel. **a** Brightfield image series of a typical pair of cells migrating on a collagen gel, adapted with permission from the work^41^. Scale bar, 30 µm. **b** CME components of the up cell as a function of time. The red and blue lines represent CMEa and CMEr, respectively, and the Spearman’s coefficient is 0.91. **c** CME components of the down cell as a function of time. The Spearman’s coefficient is 0.85, and the lag ${dN}$ is 2. **d** Average of the CME components for the up cell (green line) and the down cell (orange line). The average of CME is artificially divided into four stages according to the trends, i.e., stage I (2 – 34 min), II (36 – 52 min), III (54–70 min), and IV (72–92 min). **e** Averaged CME in each stage. Data are presented as mean ± s.d.; $* * p$ < 0.01, $* * * p$ < 0.001, *n.s*. means “not significant”; Kruskal-Wallis test. **f** Box plots of the CME components in **b**, **c** and the average of the components in **d**. The box plot indicates the mean (small square in the box), the median (black line in the box), the 25th percentile (bottom line of the box), the 75th percentile (top line of the box), and 1.5*IQR (interquartile range, bars). **g** The scatter of CMEr *vs*. CMEa. Pearson’s coefficients are 0.97 for the up cell and 0.94 for the down cell. The green and orange lines are linear fits to the corresponding scatter, obeying the functions “y = 0.91x-0.05” (R^2^ = 0.93) and “y = 0.93x-0.08” (R^2^ = 0.88), respectively.

Figure 4a shows representative time-lapse images of a pair of cells labeled “up” and “down”, apparently indicating that the two cells are migrating toward each other, as indicated by the yellow arrows. After applying the CME approach to cell morphology, we obtained the CME components of the cell pair as a function of time (Fig. 4b–d). In Fig. 4b, the CME components of the up cell show almost the same trends (Spearman’s coefficient = 0.91), i.e., the values first increase and then decrease and thus form a “stable” maximum (~0.7 for CMEa, ~0.6 for CMEr) in the time interval of about 40–70 min. However, the CME components of the down cell exhibit significant differences from those of the up cell (Fig. 4c), namely, they first fluctuate at a high level of about 0.6–0.7 for CMEa (or about 0.4–0.5 for CMEr), then start to decrease steeply from ~30 min and reach the minimum at ~50 min (~0.4 for CMEa, ~0.25 for CMEr), then gradually increase until ~70 min and finally return to the high level before decreasing. In this process, CMEa changes synchronously with CMEr, quantified by Spearman’s coefficient = 0.85. In addition, the values of CMEr are generally smaller than those of CMEa for the two cells, indicating that the angular characteristic encoded by CMEa is more sensitive than the radial characteristic encoded by CMEr when the cell responds to the same external cues from the microenvironment (see the similar results in Fig. 3b). Here, the different sensitivities may be caused by a combination of cellular intrinsic properties and extracellular cues, and which needs to be further validated by a series of experiments.

#### Symmetry and similarities in the alternating changes

In addition, the average of CMEa and CMEr vividly shows the differences between the up and the down cells, as shown in Fig. 4d. To better compare the behavioral modes of the two cells during different migration periods, we divide the average of the CME components into four stages (as indicated by the vertical dotted lines) based on their time-lapse characteristics. In stage I, i.e., 2–34 min (or stage IV, 70–92 min), the average of these two components is slightly increased (or decreased) for the down cell and is larger than the gradually increased (or decreased) average for the up cell. In contrast, in stage II, 36 – 52 min (or stage III, 54–70 min), the strongly changed average for the down cell is significantly smaller than the basically stable average for the up cell. Next, all the averages in each stage are averaged again and plotted in Fig. 4e to quantitatively validate the descriptions above. The results clearly show two types of symmetry: (i) the changing trend of the averaged CME for the down cell is almost opposite to that for the up cell; (ii) all values in stages I and II seem to be symmetric with those in stages III and IV. In addition, we also calculate the profiles of AR *vs*. time for these two cells. The results show roughly the changing trends exhibited in Fig. 4d, but these are some significant differences from the symmetry. See Supplementary Fig. 4c for more discussion.

Apart from the time-varying features of the CME, the box plot in Fig. 4f also shows that not only are all the means for the down cell slightly larger than those for the up cell but also the CME of the up cell are more sparsely distributed than those of the down cell, directly demonstrating the statistical differences in morphology between the two cells. Nevertheless, there are still some interesting similarities, such as the linear variations of CMEr *vs*. CMEa, which fit relatively well to the formulas “y = 0.91x + 0.05” (R^2^ = 0.93) and “y = 0.93x + 0.09” (R^2^ = 0.88) for the up and down cells, respectively.

#### A potential indicator of cell forces

Based on the results above, we proposed that the behavior modes encoded in morphology may embody the interactions between a pair of cells, in particular the force exerted by individual cells. In previous works^41–43^, we observed that the active tensile forces generated by migrating cells can remodel collagen fibers, which is directly verified by the phenomenon that elongated cells contribute to the formation of fiber bundles, while rounded cells don’t reorganize the surrounding collagen fibers, and in turn, the fiber bundles bridging two cells typically regulate cell migration and lead to strongly correlated motility. Therefore, the CME could be considered as a potential indicator to accurately measure how cells exert force on the surrounding environments in real-time. See more detailed analysis in the Discussion section.

### The critical transition of tumor spheroids from proliferation to invasion

In addition to the applications of the CME approach in analyzing the morphologies of cell nuclei and cell pairs, we also investigated the proliferation and invasion of three types of cell spheroids (length scale, ~ 600 µm), namely H1299 (lung cancer), MDA-MB-231 (breast cancer), and U87 (glioma tumor), based on their morphological changes analyzed by the CME approach, which differs from the complex methods including five shape parameters in our previous work^32^. The results further validate the robustness and effectiveness of this approach in analyzing the research object with different length scales.

#### Transition from proliferation to invasion detected by the CME approach

**Fig. 5:**
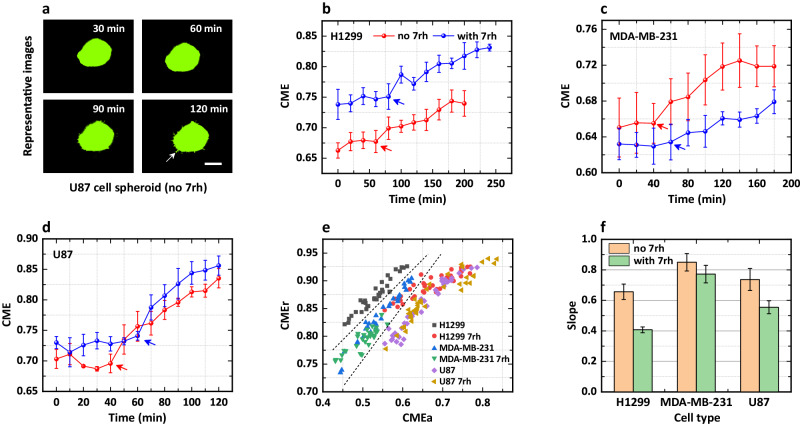
Transitions from proliferation to invasion of cell spheroids detected by the CME approach. **a** Representative fluorescence images of U87 cell spheroids without 7rh treatment. Scale bar, 300 µm. **b** CME of H1299 cells as a function of time. The red and blue lines correspond to the cases without 7rh and with 7rh treatment. **c**, **d** CME of MDA-MB-231 and U87 cells as a function of time. **e** The scatter of CMEr *vs*. CMEa for these three types of tumor spheroids. **f** Slopes of the linear fit for the six cases in **e**. Data are presented as mean ± s.e.m.; *n* = 3 independent experiments for each case; and the lag ${dN}$ is 5.

Figure 5a shows representative images of the U87 cell spheroid without 7rh (DDR1 inhibitor) treatment, which clearly shows the morphological changes of the spheroid. For example, some “fingers” formed by single cells appear at the boundary over time, as indicated by the white arrow (also see Supplementary Fig. 5 for more details on the experiments). Next, we calculated the average of CMEa and CMEr for H1299 cell spheroids (*n* = 3 independent experiments), as shown in Fig. 5b, where the average for no-7rh and with-7rh cases show similar trends, i.e., first remaining stable with minor fluctuations and then gradually increasing. The stable stage manifests that although cells begin to proliferate and lead to an expansion of the morphology of the initial spheroid, this does not affect the shape until the presence of the fingers. The fingers represent the invasion of cancer cells away from the spheroid, which may be driven by the hypoxic and acidic tumor microenvironment^44^. In addition, the transition from the “stable” to the “increase” stage (i.e., from proliferation to invasion) is earlier in the no-7rh case than that in the with-7rh case, suggesting that the DDR1 inhibitor 7rh could effectively inhibit the transition. To further validate the results, we analyzed the experimental data for MDA-MB-231 and U87 cell spheroids (*n* = 3 independent experiments for each case). Evidently, all transitions that the AR metric cannot capture (see Supplementary Figs. 4d–f and Table 1) are earlier in the no-7rh cases, indicating that the 7rh does inhibit the proliferation-invasion transition regardless of the cell type (Fig. 5c–d).

Note that all CMEs of the with-7rh case are larger than those of the no-7rh case for the H1299 cell spheroid. However, the “larger” relationship becomes “smaller” and “approximately equal” for the MDA-MB-231 and U87 cell spheroids, respectively, which may be caused by the different sizes (mean radius) of the initial cell spheroids (t = 0 min). Here, we suggest that the differences in the CME relationships have less impact on the transition results because they are mainly determined by the slopes (or inflection points) of the CME profiles rather than the magnitudes.

#### Inhibited transition derived from the CME scatter

In contrast to the results shown in Fig. 4g, the scatter of CMEr *vs.* CMEa indicates that significant differences are observed in different cell types (Fig. 5e). For the no-7rh cases, the scatter for H1299 and U87 cell spheroids are relatively distributed in the upper-left and lower-right regions, respectively, while that for MDA-MB-231 cell spheroids is located in the region sandwiched by the scatter for the other two cell types, as indicated by the dotted lines. In addition, for a given CMEa (e.g., CMEa = 0.6), H1299 cell spheroids have the largest CMEr, followed by MDA-MB-231 and U87 cell spheroids, indicating that the heterogeneity in finger length is the most pronounced in H1299 cell spheroids. When treated with 7rh, the CMEr and CMEa are significantly affected, especially for H1299 and MDA-MB-231 cell spheroids. For example, (i) the scatter of the with-7rh case for H1299 cell spheroids is significantly different from that of the no-7rh case, resulting in two separate regions (see the dotted lines); (ii) the scatter of the with-7rh case for MDA-MB-231 cell spheroids have smaller CMEr and CMEa, and forming a region with an area approximately half of that of the no-7rh case. To further explore the relationship between CMEr and CMEa, all of the scatter for the three types of cell spheroids were fitted by linear variations with the slopes plotted in Fig. 5f. The histograms clearly show that the slopes of the no-7rh cases are significantly larger than those of the with-7rh cases, for H1299 and U87 cell spheroids. At the same time, there is less difference for MDA-MB-231 cell spheroids when s.e.m. errors are considered. The results above illustrate that (i) 7rh treatment can alter the quantitative correlation of CMEr with CMEa and inhibit the invasion of single cells away from cell spheroids (see Supplementary Fig. 6); (ii) different cell types have distinct sensitivities to 7rh, which further leads to the changes in slopes of the CME profiles.

## Discussion

In this study, we have introduced an approach called “CME”, derived from a combination of morphological analysis and Shannon entropy, which allows us to analyze the angular and radial characteristics concerning cell morphology and to further explore the mechanisms of cell migration underlying morphological dynamics.

We first investigate a sub-cellular object, a cell nucleus, squeezing through a micro-structured array consisting of sequential channels and chambers, among which the channels become progressively narrower. We found that the CME of the nucleus in channels is significantly larger than that in chambers, and the changing trends of the CME vividly reflect two characteristics of the array, i.e., channels sequentially connecting to chambers and gradually narrowing channels. Therefore, it’s probably feasible to consider the CME as a powerful metric that can be directly utilized to measure the characteristics of physical cues that geometrically constrain adhesion sites^45^, such as the oriented 3D matrix or 1D lines, when direct measurements are ineffective or appropriate tools are lacking. Moreover, we quantitatively obtain the relationship between channel sizes and the angular features of the morphology for a given nucleus (Fig. 3b), which not only manifests the direct effects of physical constraints on the morphological dynamics of the nucleus but also characterizes the deformability or sensitivity to physical cues, to some extent. Furthermore, these properties can be combined to construct an identification code, such as a “fingerprint”, to signify the essential features of the cell nucleus.

Additionally, we divide the scatter of CMEr *vs*. CMEa into three clusters corresponding to three stages, i.e., migration in channels, in chambers, and entering/exiting the channels, respectively. Although it is the nuclear morphology that determines the clustering, it reflects to some extent the features of the invasiveness/migration modes of cancer cells. For example, the time cost of the nucleus is roughly estimated in performing these unique migration modes and clearly indicates a key point: the time to enter the channels is more significant than the time to exit the channels, illustrating that the cell needs more time to coordinate the intracellular signaling pathways associated with nuclear envelope stretch-sensitive proteins to better adapt to the physical constraints. The analysis above is in good agreement with the mechanisms underlying cell responses to spatial constraints reported in previous work^23^.

Except for the sub-cellular nucleus, we further studied the morphology of MCF-10A cells migrating on a thick collagen gel. We found that the changes in the morphology of the two cells that are close to each other exhibit an apparent regularity, i.e., when the morphology of one cell is compared to the rounded state, another cell elongates and deviates from the rounded state, and vice versa. From another point of view, the changes possess a high degree of symmetry in both the vertical (magnitude) and horizontal (time) axes, as indicated by the four stages in Fig. 4d, which further illustrates that an alternating mode emerges from the morphologies of the two cells and the interaction may be mediated by a communication medium, such as collagen fibers^41,46^ or biochemical factors^47^. Here, the principle of communication is similar to that used by follower cells to communicate with leader cells through adhesion-based mechanical interactions^48^. If the changed morphology is related to cell contributions to the encounter, it may refer to the forces exerted on the ECM^49,50^, the energy cost^51^, or other physical quantities.

Based on the result reported in our previous work, cell morphology is strongly correlated with the force exerted on the ECM^41^; thus, one could evaluate the characteristics of the force using the CME approach when the cell migrates in complex microenvironments, including temporal and spatial aspects. Regarding the alternating mode in Fig. 4, we argue that the down cell initially exerts a greater pulling force on the collagen fibers, and the up cell gradually increases its force after sensing the pulling force when they are far apart (from 200 to 150 μm). As the distance between them decreases (from 150 to 100 μm), the force exerted by the up cell increases and exceeds the force exerted by the down cell. Finally, when they are closer together (from 100 to 50 μm), the force exerted by the down cell becomes the major contributor again. The alternating change may allow one cell to sense or “judge” the status of another cell and further adjust its migration mode, helping to improve the efficiency of communication or correlated movement. We believe that the emerging interaction encoded in the morphology embodies to some extent the diversity of cell-cell communication, which may help to explain some collective cell migrations^52^, such as wound healing, histogenesis, and cancer cell invasion and metastasis.

Finally, we analyzed the morphology of three types of tumor cell spheroids to detect the transitions from proliferation to invasion. The results firstly illustrate that the DDR1 inhibitor 7rh can alter the quantitative correlation of CMEr with CMEa and inhibit the invasion of single cells away from the cell spheroids, which may help us to better understand malignant mammary tumors that reorient the collagen fibers to be perpendicular to the mammary gland and use these structures as “highways” for migration away from the crowded/dense regions occupied by epithelial cells^53,54^. Second, different types of tumor cells exhibit distinct sensitivities to 7rh, which were not measured by our previous method. This results in the changes in the slopes of the CME profiles^32^. Therefore, it’s essential to optimize the dosage of 7rh to control the invasion of tumor cells and avoid or reduce the development of drug resistance. In addition, 7rh can be replaced here by other biochemical factors, such as epithelial growth factor (EGF), batimastat, and glucose, to study the individual or superimposed effects of these factors on cancer. Here, the CME approach combined with the cell spheroid model may provide us with a new platform for screening and evaluating effective drug candidates in the era of personalized cancer therapy.

In summary, we proposed an approach called CME in this article, which allows us to explore the mechanisms of cell migration underlying morphological dynamics. Our results validate the utility and efficiency of the CME approach, which can be used to accurately measure the effects of geometric constraints on the cell nucleus that is viewed prevailingly as the primary source of steric hindrance for 3D invasion, in particular the positive correlation of the size of the constraint with the CME of the nucleus. Furthermore, the interactions of MCF-10A cells migrating on a thick collagen gel also emerge from the morphological changes characterized by the CME, which not only illustrates the diversity of cell-cell interactions but also emphasizes the crucial role of collagen fibers in regulating cell migration behavior. Finally, we also captured the transitions of three types of tumor cell spheroids from proliferation to invasion and further confirmed the ability of the DDR1 inhibitor 7rh to attenuate the invasiveness of cancer cells. Overall, our approach contributes to analyzing the information encoded in morphology and revealing cellular migration strategies at multiple length scales in complex microenvironments.

## Methods

### 3D collagen gel experiment

An in vitro cell migration experiment was carried out: MCF-10A cells were first obtained from China Infrastructure of Cell Line Resource (Beijing, China) and labeled with green fluorescent protein (GFP). Additionally, the culture medium used here is Dulbecco’s modified Eagle’s medium-F12 (Corning, Corning, NY), which was further supplemented with 1% penicillin/streptomycin (Corning), 5% horse serum (Gibco, Gaithersburg, MD), 20 ng/mL human EGF (Gibco), 100 ng/mL cholera toxin (Sigma-Aldrich, St. Louis, MO), 0.5 mg/mL hydrocortisone (Sigma-Aldrich), and 10 mg/mL insulin (Roche Diagnostics, Basel, Switzerland). Next, type I collagen extracted from rat tail tendon (Corning) was diluted and neutralized to pH ~ 7.1. The collagen solution was then spread on the substrate of a Petri dish and incubated at 37 ^o^C for 30 min until it polymerized into a 3D matrix with a concentration of 2 mg/mL and a thickness of ~ 2 mm. Then, 0.5 mL of the cell suspension was dropped on top of the collagen gel and formed a randomly distributed cell population with a low cell density of 10^4^ cells/cm^2^ after incubation for 2 h. Finally, a confocal laser scanning microscope and an automatic inverted fluorescence microscope (Nikon Ti-E, Tokyo, Japan) were used to obtain the time-lapse images of the cells with a sampling time of 2 min. See our previous works^41,42^ for further details of the experiment.

### Data regarding cell spheroid

The experiment was previously performed using three types of tumor cells, including U87 (glioma tumor) cells, H1299 (lung cancer) cells, and MDA-MB-231 (invasive breast cancer) cells, all of which were first labeled with a green fluorescent protein (GFP). A cell suspension was then prepared at a density of 1.0 × 10^4^ cells/mL and seeded into an ultra-low attachment (ULA) plate with a 96-well round bottom, where a cell spheroid formed after 96 h. Next, the spheroid was transferred to a new 96-well flat bottom ULA plate containing culture medium with a collagen concentration of 2 mg/mL, where the spheroid was imaged by a CCD camera (Neo 5.5 sCMOS, Andor, USD) for further analysis. See our previous work^32^ for more details of the experiment.

### Data regarding micro-structured array

A micro-structured array consisting of sequential channels and chambers was designed and fabricated using a combination of soft lithography and polydimethylsiloxane (PDMS), in which the channel is 20 µm length, 3.7 µm height, and has a decreasing width from 11.2 to 1.7 µm. To better observe and analyze the dynamic process of invasive MDA-MB-231 cells squeezing through the array, cell nuclei were stained with 1.5 µg/mL Hoechst 33342 (in red) and imaged with a sampling time of 0.2 min. Note that the images of cell nuclei analyzed in this study were taken with permission from the attached videos of the work^22^.

### Image processing and boundary extraction

After obtaining the time-lapse microscopy image, we first manually extract the morphology of a research object (e.g., cell nuclei, single cells, or cell spheroids) from complicated backgrounds using the basic operations (including “Brightness/Contrast”, “Threshold”, “Find Edges”, “Fill Holes”, and “Outline”) in ImageJ software. Second, the extracted morphology is transformed into a grayscale image, and further, the noises contained in the image are reduced by calling the built-in functions (including “rgb2gray”, “fspecial”, “imfilter”, “graythresh”, “imbinarize”, and “imfill”) in MATLAB software (MathWorks, USA). Then, Otsu’s method^55^ is introduced to automatically transform the grayscale image into a binary one containing only two grayscale levels (0 and 255). Subsequently, the “imfill” function is called to fill the holes in the binarized image, resulting in many connected domains, among which the domain with the largest area is identified as the main body of the research object, i.e., the 2D morphology. Finally, the centroid and the boundary of the morphology are naturally determined by the “regionprops” function in the Cartesian coordinate system (CCS) for further analysis (see Fig. 1a).

### Morphological analysis

To better understand the process of developing the CME approach, we first map the morphology in the CCS onto the polar coordinate system (PCS) and move the centroid of the morphology to the origin of the PCS. Then, we obtain the coordinates $\left({r}_{i},{\theta }_{i}\right)$ of the boundary for $i$ = 1, 2, $\cdots$, and $N$, which accurately describe the shape of the research object. Here, the subscript $i$ denotes the numerical order of each point on the boundary. Since the boundary exactly contains some “burrs” that are mainly caused by system noises, it is essential to define a number lag ${dN}$ for extracting a new boundary from the original boundary, i.e., the coordinates $({r}_{j},{\theta }_{j})$ are selected to form a new boundary ${\bf{M}}=({r}_{j},{\theta }_{j})$ where $j$ = ${dN}$, $2\cdot {dN}$,$\cdots$, and floor$\left(N/{dN}\right)\cdot {dN}$. See Supplementary Fig. 2 for the detailed discussion of how to determine the ${dN}$. Although the new boundary discards some burrs, it still represents the main shape of the research object. Next, the displacements between any two consecutive points are computed by $\Delta {\bf{M}}={{\bf{M}}}_{j}-{{\bf{M}}}_{j-1}$, which can also be represented by two terms, i.e., radial $\Delta {r}_{j}$ and angular $\Delta {\theta }_{j}$ components. Subsequently, the PDFs of the two components are derived from their normalized statistical histograms, i.e., $p\left(\Delta r\right)$ and $p\left(\Delta \theta \right)$, as shown in Fig. 1b.

### CME approach derived from Shannon entropy

Inspired by the relationship above, we further introduce Shannon entropy to develop a robust description method for quantifying the changes in morphology (see flowchart in Fig. 1c). Entropy is an extensively used concept in thermodynamics and is typically used to describe the degree of order or randomness in the states of molecules. It was first introduced by C. E. Shannon in 1948 to describe the “uncertainty” in information sources^56^. Thus, it is also called “Shannon entropy” when used in information theory. Actually, entropy is technically quite difficult to compute reliably for continuous variables^57^. However, for a random event with discrete probabilities of occurrence ${p}_{1}$,$\,{p}_{2}$, …,$\,{p}_{n}$, the corresponding entropy can be easily derived from the following formula:

1 $$H=-\mathop{\sum }\limits_{i=1}^{n}{p}_{i}\log 2\left({p}_{i}\right)$$

where $n$ is the total number of events that possibly occur, ${p}_{i}$ is the probability of each event occurring, and $\log 2$() is the logarithmic function with a base of 2. The Shannon entropy not only measures how much “choice” is involved in selecting an event, but also indicates how “uncertain” the outcome of the event might be. According to the properties of the logarithmic function above (Eq. 1), it is not difficult to deduce that the entropy $H$ will be maximum when the probabilities ${p}_{i}$ are identical to each other, i.e., ${p}_{i}=1/n$, meaning that one cannot judge which event is most likely to occur^42^. Since the entropy above is strongly correlated with the number of events, we further normalize all entropies by dividing the maximum $H$ to eliminate this correlation, resulting in all entropies (denoted by $\widetilde{H}$) being rescaled to a narrow interval of [0,1]. Next, we further replace the ${p}_{i}$ by $p\left(\Delta \theta \right)$ or $p\left(\Delta r\right)$ separately, and obtain the $\widetilde{H}$ for each component. According to the physical meaning of Shannon entropy, it is evident that the more regular (or irregular) the shape of the object, the closer the corresponding $\widetilde{H}$ is to 0 (or 1). To avoid confusion caused by abbreviations, we use “CME” to denote the entropy $\widetilde{H}$ that correlates with the morphology of the object.

### Calculation of aspect ratio

To effectively compare the results obtained by CME and aspect ratio (AR), we first process the original images using the scripts written in Matlab, then export these images to fit the object in each image with an ellipse using the “Fit ellipse” function in ImageJ and obtain the length of the major and minor axes of the ellipse, and finally calculate the AR by dividing the length of the major axis by the length of the minor axis.

### Cross-correlation

The cross-correlation *C*_*r*θ_ (*n*) between the radial $\Delta {r}_{j}$ and angular $\Delta {\theta }_{j}$ components is defined as ${C}_{r\theta }\left(n\right)=\frac{{\left\langle \left(\Delta {r}_{j}-\bar{\Delta r}\right)\left(\Delta {\theta }_{j+n}-\bar{\Delta \theta }\right)\right\rangle }_{j}}{\sqrt{{\sigma }_{r}^{2}}\sqrt{{\sigma }_{\theta }^{2}}}$, where $\bar{\Delta r}={\langle \Delta {r}_{j}\rangle }_{j}$ and $\bar{\Delta \theta }={\langle \Delta {\theta }_{j}\rangle }_{j}$, are the averages, and ${\sigma }_{r}^{2}{=\langle {(\Delta {r}_{j}-\bar{\Delta r})}^{2}\rangle }_{j}$, and ${\sigma }_{\theta }^{2}{=\left\langle {\left(\Delta {\theta }_{j}-\bar{\Delta \theta }\right)}^{2}\right\rangle }_{j}$, are the corresponding variances of the components.

### Statistical analysis

Statistical analysis was performed with custom MATLAB (R2018b, USA). If the data meet the criteria of normality and equal variance, parametric tests are used, i.e., *t*-test for two groups and ANOVA (analysis of variance) for more than two groups. If the data do not meet the criteria, then a bijective transformation is introduced and used to process the data. If the data meet the criteria after processing, then parametric tests are used, otherwise, non-parametric tests are applied, i.e., Wilcoxon rank sum for two groups and Kruskal-Wallis for more than two groups. It should be noted that normality and equal variance are tested by the “llillietest” (i.e., Lilliefors test) and “vartestn” (i.e., Bartlett test) functions in MATLAB, respectively, while the “acos” function is used in this work to perform a bijective transformation. Differences are significant at the 95% confidence level (two-tailed). There are three levels of significance: $* p$ < 0.05; $* * p$ < 0.01; $* * * p$ < 0.001, according to the standard Michelin Guide scale.

### Correlation coefficients

The correlation coefficient reflects the degree of correlation between two variables. If the data are continuous numerical variables, and both satisfy normality (or possess obvious single peaks), the Pearson coefficient is preferred, and if the data do not satisfy the normality after transformation, the Spearman or Kendall coefficients are optional. See more details in the work^42^.

### Reporting summary

Further information on research design is available in the Nature Research Reporting Summary linked to this article.

### Supplementary information

Supplemental Material

Reporting Summary

## Data Availability

The data used in this study is available from previous publications referenced in the text^22,41,58^: 10.1016/j.bpj.2015.07.025^22^, 10.1002/anie.202016084^41^, 10.1038/s41598-018-30408-7^58^ (with permissions). Data regarding tumor spheroids of this study is available from the corresponding author upon request.

## Code availability

The custom codes used to calculate the CME approach in this study can be found at https://github.com/YanpingRoy/Morphological-Entropy.git.
