# Supplementary material for: Morphological entropy encodes cellular migration strategies on multiple length scales
Source: NPJ Syst Biol Appl. 2024 Mar 7;10:26. doi: 10.1038/s41540-024-00353-5 (PMC10920856; doi:10.1038/s41540-024-00353-5)
Supplement: Supplementary file 1 — Supplemental Material [file 41540_2024_353_MOESM1_ESM.pdf]

Supplementary Material

Morphological Entropy Encodes Cellular Migration Strategies on Multiple Length Scales

Yanping Liu[1,2], Yang Jiao[3,4], Qihui Fan[5], Xinwei Li[1,2], Zhichao Liu[1,2], Dui Qin[1,2], Jun Hu[6], Liyu Liu[7], Jianwei Shuai[8,9,10,*], Zhangyong Li[1,2,*].

[1] Department of Biomedical Engineering and [2] Chongqing Key Laboratory of Big Data for Bio Intelligence, Chongqing University of Posts and Telecommunications, Chongqing 400065, China

[3] Materials Science and Engineering and [4] Department of Physics, Arizona State University, Tempe, Arizona

[5] Beijing National Laboratory for Condensed Matter Physics and CAS Key Laboratory of Soft Matter Physics, Institute of Physics, Chinese Academy of Sciences, Beijing 100190, China

[6] Department of Neurology, Southwest Hospital, Army Medical University, Chongqing 400038, China

[7] Chongqing Key Laboratory of Soft Condensed Matter Physics and Smart Materials, College of Physics, Chongqing University, Chongqing 401331, China

[8] Department of Physics and [9] Fujian Provincial Key Laboratory for Soft Functional Materials Research, Xiamen University, Xiamen 361005, China

[10] Wenzhou Institute, University of Chinese Academy of Sciences, Wenzhou 325000, China

*Corresponding authors: jianweishuai@xmu.edu.cn, lizy@cqupt.edu.cn

Contents:

1. Exploration of CME from small differences in morphology and the effect of number lag dN

1.1 CME captures small differences in lamellipodia morphology

1.2 Number lag dN has little effect on the information encoded in CME

2. Cross-correlation between the radial displacement Δr_j and angular displacement $\Delta\theta_j$

3. Relationship between CME and aspect ratio (AR) that measures the elongation of the cell

3.1 AR of the cell nuclei, single cells, and tumor spheroids

3.2 CME as a measure of elongation and irregularity

4. Further details of the tumor spheroid experiments

4.1 Time-lapse images of three types of tumor spheroids

4.2 Transition time from proliferation to invasion obtained from the CME profiles

Supporting References

1. Exploration of CME from small differences in morphology and the effect of number lag dN

1.1 CME captures small differences in lamellipodia morphology

In the main text, we have clarified the biophysical interpretations of the CME approach by analyzing two types of representative single-cell migration modes, i.e., amoeboid and mesenchymal motilities. The results clearly illustrate the relationship between angular (or radial) features and CMEa (or CMEr), thus allowing us to capture the main morphology of a given research object according to CME components.

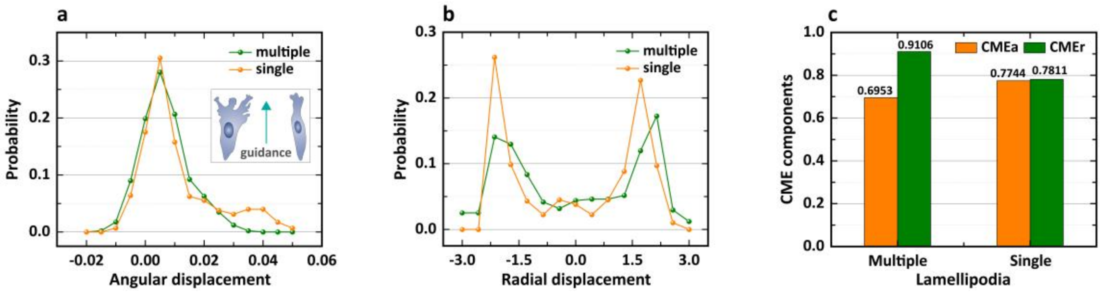

Supplementary Figure 1. Application of the CME approach to the analysis of cell migration lamellipodia. (**a**) PDFs of the angular displacement of cell morphology. The inset shows representative multiple and single lamellipodia, adapted from the work [1] with permission. (**b**) PDFs of the radial displacement of cell morphology. (**c**) CME components of the two lamellipodia types.

To further verify the performance of the CME approach in distinguishing the subtle morphological changes, we again analyze two types of representative morphologies during cell migration, i.e., multiple and single lamellipodia (see the inset of Fig. S1a). Overall, the PDFs of angular displacement for the two types of lamellipodia show similar trends, but with subtle differences, e.g., the probability values for single lamellipodia are larger than those for multiple lamellipodia in the interval of 0.02 ~ 0.05 (Fig. S1a), which directly leads to a wider PDF for the former with a larger CMEa of 0.7744 and a narrower PDF for the latter with a smaller CMEa of 0.6953 (see orange bars in Fig. S1c). Similarly, the PDF of the radial displacement for multiple lamellipodia is relatively broader due to the smaller values at the peaks (Fig. S1b), corresponding to a larger CMEr of 0.9106 compared to a smaller CMEr of 0.7811 (see green bars in Fig. S1c). The above results not only demonstrate the quantitative differences between multiple and single lamellipodia but also further validate the superior ability of the CME metric to analyze the changes in morphological features of a given object, including significant and non-significant differences.

1.2 Number lag dN has little effect on the information encoded in the CME

In the development of the CME approach, it is important to define a number lag dN for extracting a new boundary from the original boundary of the morphology. According to the definition of dN, the new boundary is identical to the original one when $dN = 1$, and more points forming the original boundary are filtered with the increase of dN. As a result, a smaller dN corresponds to more points that may contain some noise resulting from the imaging system in the experiment. In comparison, a larger dN corresponds to fewer points, which may omit some crucial features of

the morphology regarding cell migration. Thus, how to determine the value of dN becomes an important issue.

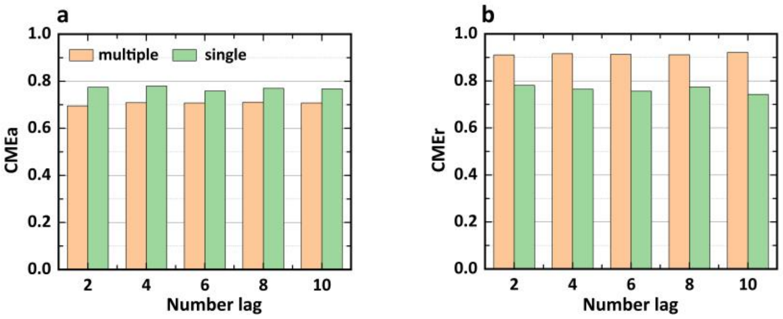

Supplementary Figure 2. Effects of number lag dN on CME components for multiple and single lamellipodia Number lag dN has almost no effect on CMEa (**a**) and CMEr (**b**).

To better address this issue, we again analyze the representative images of cell morphology with multiple and single lamellipodia [1] (see the inset of Fig. S1a) and obtain the final results (Fig. S2). It is clear that as dN increases from 2 to 10, the CMEa of multiple and single lamellipodia are almost stable at 0.71 and 0.77, respectively (Fig. S2a). In addition, the CMEr of the two types of lamellipodia also remains basically stable at 0.91 and 0.76, respectively (Fig. S2b). The above results indicate that the number lag dN in a small range (2 ~ 10) almost does not affect the CME components (or encoded information). Thus, one could define dN according to the following criteria: 1) if the morphology (image) that needs to be analyzed has been processed by some operations, e.g., smoothing and filtering, or in other words it has little noise, it is better to choose a small dN to consider more features; 2) if the morphology is still noisy even after denoising, it is necessary to define a larger dN to discard some features that are strongly affected by the system noise. It should be noted that we only study the effects of dN in a small range on the CME components because the larger dN will definitely correspond to a value of CME that deviates from the intrinsic value and will gradually decrease the accuracy of the CME approach.

2. Cross-correlation between the radial displacement Δr_j and angular displacement $\Delta\theta_j$

In the main text, we examine the scatter of CMEr vs. CMEa and find that the CMEr is fundamentally and positively correlated with the CMEa. To explore the potential causality of this correlation, we again analyze tumor spheroids from different independent experiments by tracing back to the calculation of the CME. For the procedures described in the main text, we mainly focus on the radial Δr_j and angular $\Delta\theta_j$ displacements and further compute the cross-correlation between them (see Fig. S3). None of the results show an obvious changing trend, but fluctuate with increasing of lag. Nevertheless, most of the values are positive (<0.2) (see panels a, c, e, and f), indicating that the Δr_j and $\Delta\theta_j$ are weakly correlated. Therefore, it can be inferred that the corresponding distributions $p(\Delta r)$ and $p(\Delta\theta)$ are correlated to some extent, or the former is slightly similar to the latter, and consequently, the CMEr is also related to the CMEa. In addition, it

should be noted that the negative values (see panels b and d) would change the distributions and then affect the correlation between the CME components.

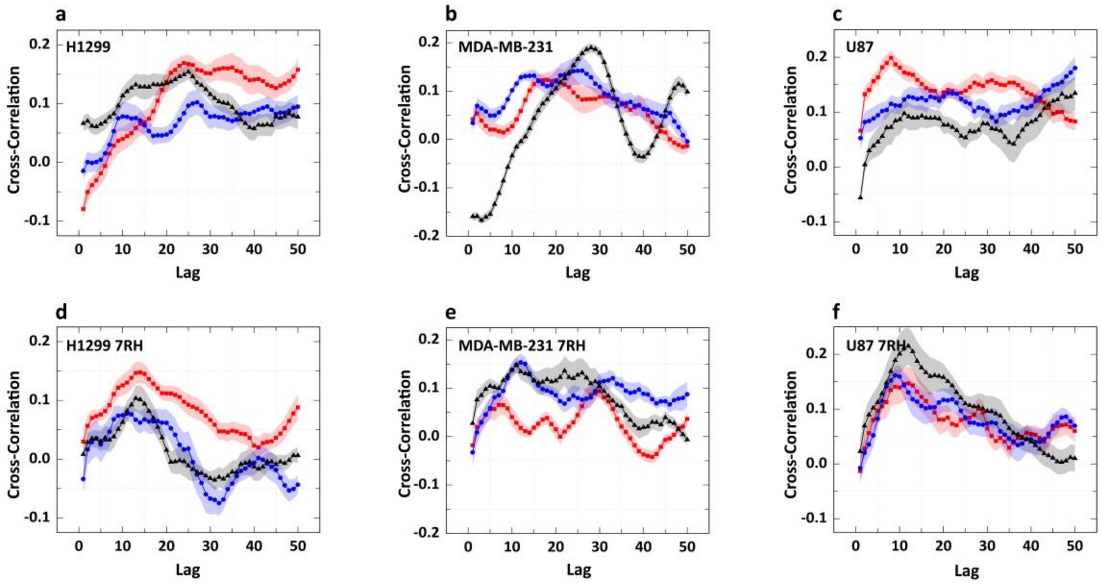

Supplementary Figure 3. Cross-correlation between the radial Δr_j and angular $\Delta\theta_j$ components. Cross-correlation for H1299 tumor spheroids without 7rh treatment (**a**) and with 7rh treatment (**d**). (**b**, **e**) Cross-correlation for MDA-MB-231 tumor spheroids. (**c**, **f**) Cross-correlation for U87 tumor spheroids. Data are presented as mean ± s.e.m.; n is the number of sampled images of spheroids in each independent experiment, indicated by colored lines.

3. Relationship between CME and aspect ratio (AR) that measures the elongation of the cell

3.1 AR of the cell nuclei, single cells, and tumor spheroids

In this work, we develop a CME approach and use it to study the morphological changes of cell nuclei, single cells, and tumor spheroids. The results presented in the main text clearly illustrate three aspects: i) the changes of cell nuclei in geometrical confinements; ii) the correlated changes of a cell pair on top of a 3D collagen gel; and iii) the transition of tumor spheroids from proliferation to invasion. Obviously, the above results are correlated with the elongation of the research objects, especially the correlation of elongation with the morphological dynamics of cell nuclei in the narrow channel. Therefore, we further measure the elongation of the research objects using a classical method called "aspect ratio" (AR) to verify the superior ability of the CME approach (Fig. S4a). Fig. S4b shows a clear AR profile with four peaks and three valleys, which is very similar to that in Fig. 3c obtained by the CME approach. In addition, the AR profiles (Fig. S4c) for a pair of cells migrating on top of a 3D collagen gel also show similar changing trends to the results shown in Fig. 4d. Nevertheless, there are significant differences between the profiles obtained by the CME and AR approaches, especially the asymmetry along the vertical and the horizontal axes. More importantly, all of the AR profiles for three types of tumor spheroids show non-monotonic behavior

(Fig. S4d-f), i.e., they don't significantly increase or decrease with increasing time but remain stable, indicating that the elongation measured by the AR metric for tumor spheroids does not change with time. In fact, the stable trends are not consistent with the process of the formation of some small "fingers" at the boundary of the spheroids, which is well quantified by the gradually increasing trends shown in Fig. 5.

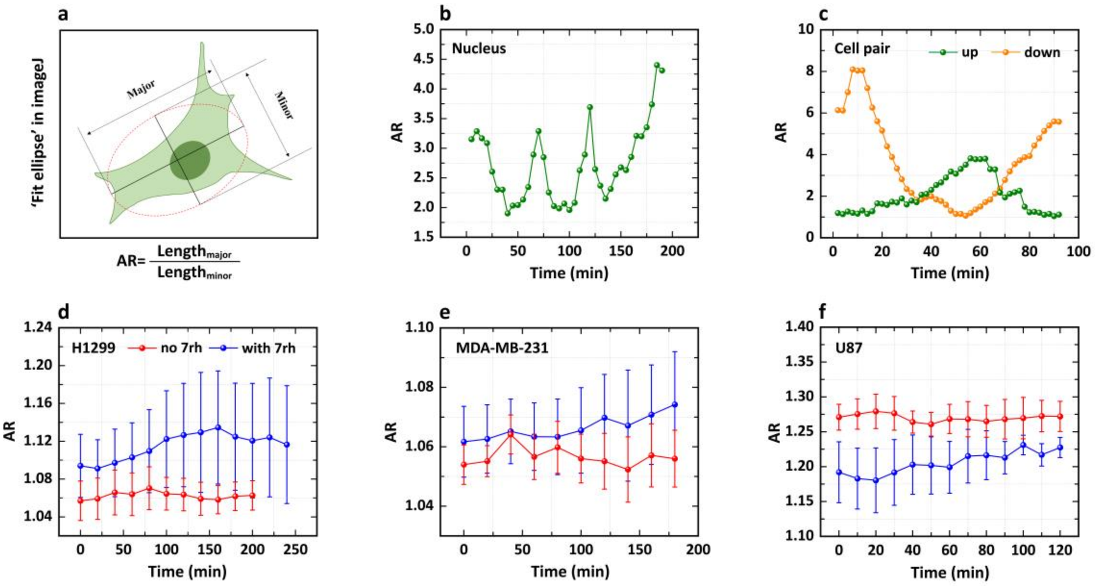

Supplementary Figure 4. AR of the cell nuclei, single cells, and tumor spheroids. (**a**) Principle of AR calculation, adapted from the work [2] with permission. (**b**) AR of the nucleus squeezing through a narrow channel. (**c**) AR of a pair of cells migrating on top of a 3D collagen gel. AR of H1299 (**d**), MDA-MB-231 (**e**) and U87 (**f**) cell spheroids. Note that the data analyzed above correspond to the three types of experimental data. Data are presented as mean ± s.e.m.; n=3 independent experiments for each case.

3.2 CME as a measure of elongation and irregularity

Regarding the difference in performance between the CME and AR approaches, we further verify the effectiveness of the CME approach by combining the artificial judgment of the research object. Simply, we randomly select two tumor spheroids in each case according to the "almost equal" values of AR (see Table S1). It's easy to see that the CME values are usually different due to the irregular "fingers" at the boundary of morphology. For example, the first spheroid of the H1299 case has few "fingers" and has a smaller CME of 0.7363, while the second spheroid has more "fingers" and has a larger CME of 0.7685. In addition to this case, five other cases also show similar relationships that are consistent with the biophysical interpretations of the CME metric presented in the main text, i.e., the CME components describe the heterogeneity (or irregularity) of the angular and radial features, respectively. However, according to the definition of the AR metric, the AR of an irregular shape is actually equal to the AR of the corresponding "fitted" ellipse, such as the green cell and the red ellipse shown in Fig. S4a. Obviously, the AR metric does not capture the irregular features contained in the morphology.

Taken together, we could directly conclude two points as follows: i) the AR metric is able to quantify the elongation of a more regular object (e.g., an ellipse or cell nucleus), but shows the weaker ability to measure the irregular features of a given object (e.g., the tumor spheroids); ii) the CME approach can quantify not only the elongation but also the irregularity of the object, thus possessing the "duality" in analyzing the changes in morphology.

Supplementary Table 1. Tumor spheroids with "almost equal" values of AR have different values of CME.

Type	Image	AR	CME	Type	Image	AR	CME
H1299	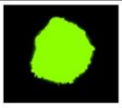	1.0922	0.7363	H1299 7RH	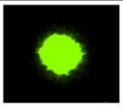	1.0849	0.8045
	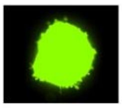	1.0924	0.7685		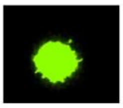	1.0844	0.8322
MDA-MB-231	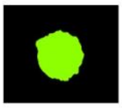	1.0671	0.7017	MDA-MB-231 7RH	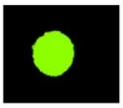	1.0877	0.6636
	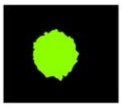	1.0675	0.7633		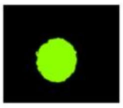	1.0878	0.6717
U87	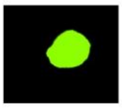	1.2324	0.6840	U87 7RH	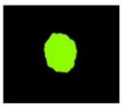	1.2436	0.7280
	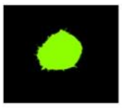	1.2326	0.8363		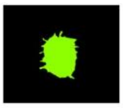	1.2434	0.8874

4. Further details of the tumor spheroid experiments

4.1 Time-lapse images of three types of cell spheroids

To visually observe the changes in the morphology of the cell spheroids, we displayed the representative images, as shown in Fig. S5. It's clear that with time, more "fingers" appear at the boundary of the cell spheroid, indicating the process from proliferation to invasion. Note that H1299 cell spheroids without 7rh treatment are larger than those with 7rh treatment (see 1st and 2nd rows), which is mainly caused by the different sizes of the spheroids at the initial time point. As shown in the main text, the different size has almost no effect on the transition from proliferation to invasion, since the transition time is only related to the presence of fingers. Of course, the larger size (more cells) may influence the internal environment of the tumor spheroid to some extent, e.g., earlier presence of hypoxic and acidic conditions, which may promote the transition. The detailed study of the effects of the internal environment is beyond the scope of this article and needs to be explored in the future.

In contrast to the different sizes for H1299 cell spheroids, the sizes of cell spheroids without 7rh are almost identical to those with 7rh for MDA-MB-231 and U87 (see 3rd ~ 6th rows). Although the fingers for the MDA-MB-231 cell spheroids are not significant when compared to those for the U87 cell spheroids, it is evident that the non-significant changes are captured by the CME metric, as shown in Fig. 5c of the main text, which also further illustrates the utility and efficiency of the CME approach.

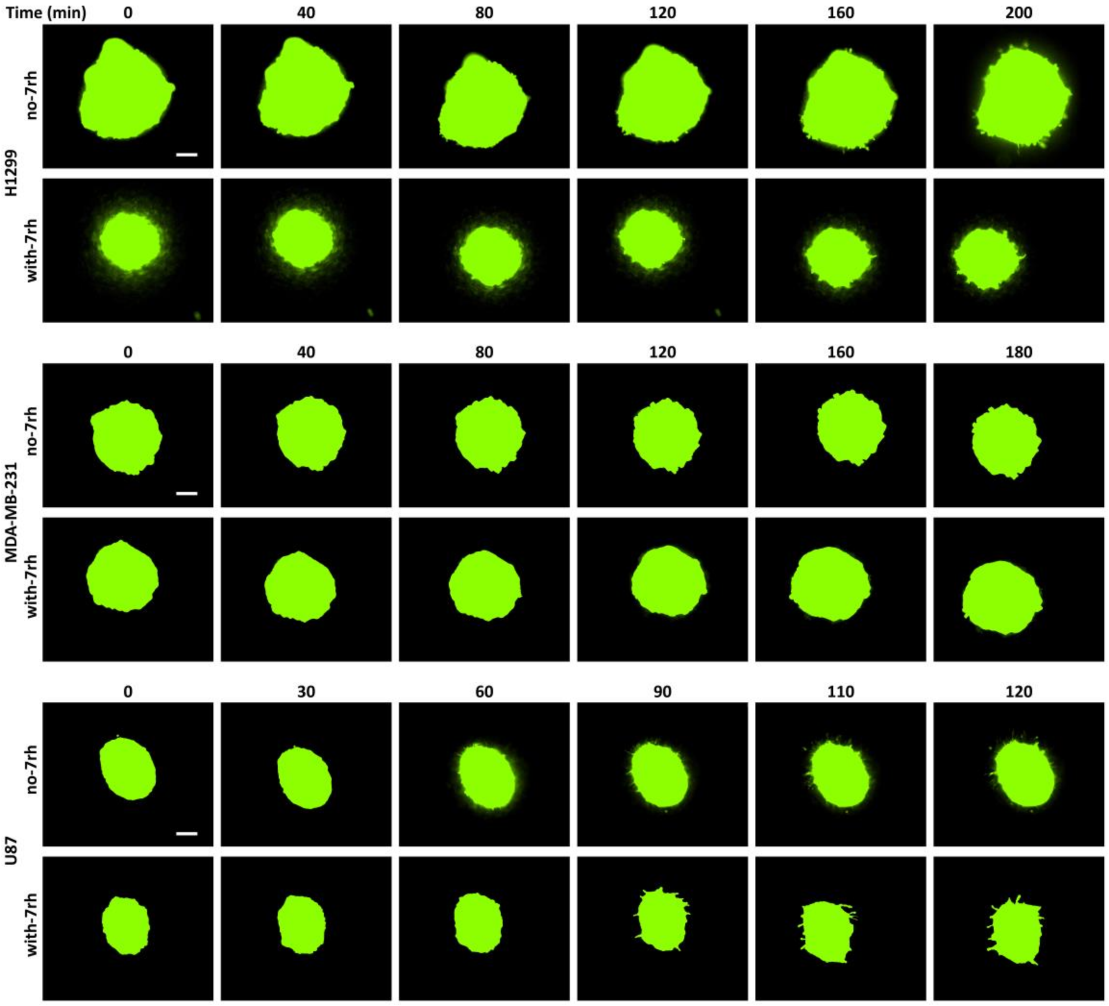

Supplementary Figure 5. Representative time-lapse images of H1299, MDA-MB-231, and U87 cell spheroids. All cells tested were labeled with a green fluorescent protein (GFP), and GFP was transfected into cells of three cell lines by the lentiviral infection method. Note that the sampling time interval is 20, 20, and 10 min for H1299, MDA-MB-231, and U87 cell spheroids, respectively. Images of U87 without 7rh (5th row) are adapted from the work [3] with permission. Scale bar, 200 μm.

4.2 Transition time from proliferation to invasion obtained from the CME profiles

According to the CME profiles in Fig. 5 of the main text, we artificially determine the inflection points and calculate the transition times as shown in Fig. S6. Overall, all transition times for the

with-7rh case are longer than those for the no-7rh case, indicating that DDR1 inhibitor 7rh attenuates single cell invasion. In addition, the transition times for MDA-MB-231 cell spheroids are close to those for U87 cell spheroids, indicating that the ability to transition is similar to each other, possibly due to the coarse sampling time. Furthermore, all the corresponding transition times for MDA-MB-231 and U87 cell spheroids are smaller than those for H1299 cell spheroids, which means that H1299 cells have a weaker invasion ability. It should be noted that there are some differences between the results in Fig. S6 and those reported in the paper [3] to some extent, which are mainly due to the following aspects: 1) less experimental data are analyzed due to the difficulty in obtaining data; 2) the CME approach is utilized to characterize the global angular and radial features of the morphology, different from the most significant fingers studied in the work [3].

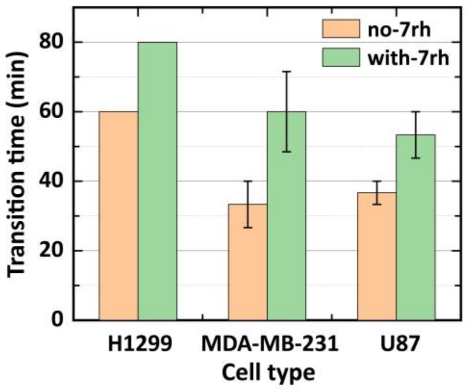

Supplementary Figure 6. Transition times of H1299, MDA-MB-231, and U87 cell spheroids from proliferation to invasion. Data are presented as mean ± s.e.m., n=3 independent experiments. Note that there are no error bars for the H1299 cell spheroids because the extracted time from the three independent experiments is identical.

Supplementary References

1 Petrie, R. J., Doyle, A. D. & Yamada, K. M. Random versus directionally persistent cell migration. *Nat. Rev. Mol. Cell Biol.* **10**, 538-549 (2009). https://doi.org/10.1038/nrm2729

2 Mohd Razali, N. A., Lin, W.-C., Norzain, N. A. & Yu, Z.-W. Controlling cell elongation and orientation by using microstructural nanofibre scaffolds for accelerating tissue regeneration. *Materials Science and Engineering: C* **128**, 112321 (2021). https://doi.org/https://doi.org/10.1016/j.msec.2021.112321

3 He, Y. *et al.* Morphological quantification of proliferation-to-invasion transition in tumor spheroids. *Biochim. Biophys. Acta-Gen. Subj.* **1864**, 6 (2020). https://doi.org/10.1016/j.bbagen.2019.129460
